# Response to “Letter to the editor: Labral calcification plays a key role in hip pain and symptoms in femoroacetabular impingement”

**DOI:** 10.1186/s13018-020-01799-z

**Published:** 2020-07-22

**Authors:** Giovanni Trisolino, Marta Favero, Dante Dallari, Enrico Tassinari, Francesco Traina, Miguel Otero, Steven R. Goldring, Mary B. Goldring, Chiara Carubbi, Roberta Ramonda, Stefano Stilli, Brunella Grigolo, Eleonora Olivotto

**Affiliations:** 1 Pediatric Orthopedic and Traumatology, IRCCS Istituto Ortopedico Rizzoli, via G.C.Pupilli 1, 40136 Bologna, Italy; 2 Rheumatology Unit, Department of Medicine (DIMED), University Hospital of Padova, Via Giustiniani 2, 35128 Padua, Italy; 3 Reconstructive Orthopaedic Surgery Innovative Techniques - Musculoskeletal Tissue Bank; Revision surgery of hip prosthesis and development of new implants, IRCCS Istituto Ortopedico Rizzoli, via G.C.Pupilli 1, 40136 Bologna, Italy; 4 Orthopaedic-Traumatology and Prosthetic surgery and revisions of hip and knee implants, IRCCS Istituto Ortopedico Rizzoli, via G.C.Pupilli 1, 40136 Bologna, Italy; 5 HSS Research Institute, Hospital for Special Surgery, 535 E 70th St, New York, NY 10021 USA; 6 RAMSES Laboratory, RIT Department, IRCCS Istituto Ortopedico Rizzoli, via di Barbiano 1/10, 40136 Bologna, Italy

Dear Editor,

We are writing in reply to Dr. Zhong’s comments regarding our recent publication [1]. While we appreciate the discussion and feedback, we are also concerned that some of these comments are somewhat vague and may be misleading. Our specific replies to Dr. Zhong’s comments are appended below.

We thank Drs. Zhong and Ouyang for reading our work and for the discussion. We enclosed a point-by-point response to their questions.

Firstly, we acknowledge that the lack of samples of healthy controls is an important limitation to our paper. Unfortunately, the Italian law and the IOR ethical committee do not allow for tissue sampling from donors for research purposes. So, we can obtain fresh samples from donors only in case they were not used during the operation after assignment. The same limitation was mentioned in a previous paper from our group, concerning fresh menisci from donors [2]. Concerning the present study, this issue is further complicated by the fact that samples of labrum from donors should be matched by age and sex with samples from patients with FAI. In our opinion, age-matching is a critical aspect since we believe that comparing labrum from young FAI patients with “healthy” aged donors could be misleading.

We should point that the FAI patients enrolled in our study had a mean age of 33 years as opposed to 62 years in the work mentioned by Drs. Zhong and Ouyang [3]. These significant differences in age should be considered when trying to compare “healthy” and “FAI” labral tissues. The “physiological process” hypothesized by the commenters could be due to aging, so it should not be present in young adults. More importantly, as clearly stated in the abstract and throughout the manuscript, our work focused on the association between labrum and synovium (and other joint tissues) in patients with femoroacetabular impingement (FAI) and the impact of these changes on surgical outcomes. Therefore, comparisons with healthy tissues were out of the scope of this study. Nonetheless, our research is ongoing (we have had just some delay due to the COVID-19 pandemic); these aspects will be presented in an upcoming article.

Concerning the second comment, Dr. Zhong’s speculation is biased by the erroneous assumption that FAI (and consequently the labral tear) is limited to the anterosuperior region. We have extensively explained in the manuscript that we collected 6 cases with isolated CAM deformity, 3 cases with isolated Pincer, and 12 mixed cases. We found that labral damage was not always and not only limited to the anterior-superior region but sometimes involved the entire labrum. However, the specimens were taken by debrided labral tissues from the most damaged labral zone. We could not collect multiple random samples from apparently “healthy” zones of labrum because it was unfeasible from an ethical point of view. Therefore, we cannot assure that the histopathological features of the specimen correspond with the characteristics of the entire labrum.

Hubert et al. [4] stained all samples with von Kossa to confirm “calcium-phosphate deposition,” and alizarin red is well-established alternative to stain “calcium-phosphate deposition” in joint tissues. We agree that the detection of BCP crystals is particularly difficult, and to distinguish BCP and CPPD, we require additional measurements, like polarized or transmission electron microscopy [5, 6]. Our ongoing studies (in collaboration with Dr. Oliviero [7]) aim to further characterize the nature of calcium deposits in FAI and OA patients, in line with Dr. Favero’s (co-author in this study) expertise and research interest. We hope to report these findings soon.

Previous study stated that the amount of calcification in the labrum instead of histological degeneration grade had significant influence on the preoperative Harris Hip Score (HHS) in patients with end-stage OA [3, 4]. Therefore, how much amount of calcium crystal deposition in the labrum produce adverse effect on hip function in patients with FAI needs further study.

This is already mentioned in the Discussion “higher local concentration of calcium crystal deposition in the labrum could lead to increased release of nociceptor stimulating substances within the fibrocartilage tissue, which densely innervated” along with pertinent references. Unfortunately, the study is not powered to establish a statistically significant threshold of calcium crystals deposition in the labrum.

Sincerely,

Giovanni Trisolino,

Marta Favero,

Enrico Tassinari,

Eleonora Olivotto

## References

[1] Trisolino G, Favero M, Dallari D, Tassinari E, Traina F, Otero M (2020). Labral calcification plays a key role in hip pain and symptoms in femoroacetabular impingement. J OrthopSurg Res..

[2] Battistelli M, Favero M, Burini D, Trisolino G, Dallari D, De Franceschi L (2019). Morphological and ultrastructural analysis of normal, injured and osteoarthritic human knee menisci. Eur J Histochem..

[3] Hawellek T, Hubert J, Hischke S, Krause M, Bertrand J, Schmidt BC (2018). Calcification of the acetabular labrum of the hip: prevalence in the general population and relation to hip articular cartilage and fibrocartilage degeneration. Arthritis ResTher..

[4] Hubert J, Hawellek T, Moe M, Hischke S, Krause M, Rolvien T (2018). Labral calcification in end-stage osteoarthritis of the hip correlates with pain and clinical function. J Orthop Res..

[5] Yavorskyy A, Hernandez-Santana A, McCarthy G, McMahon G (2008). Detection of calcium phosphate crystals in the joint fluid of patients with osteoarthritis – analytical approaches and challenges. Analyst.

[6] Frallonardo P, Oliviero F, Peruzzo L, Tauro L, Scanu A, Galozzi P, et al.. Detection of calcium crystals in knee osteoarthritis synovial fluid: a comparison between polarized light and scanning electron microscopy. J of Clin Rheumatol 2018 October 2016 - Volume 22 - Issue 7 - p 369-371. 10.1097/RHU.0000000000000416 27660935

[7] Oliviero F, Galozzi P, Ramonda R, LeitedeOliveiraF, Schiavon F, Scanu A et al. Unusual findings in synovial fluid analysis: a review. Ann Clin Lab Sci. May-June 2017;47(3):253-259). 28667024

